# A Systematic Review Examining the Approaches Used to Estimate Interindividual Differences in Trainability and Classify Individual Responses to Exercise Training

**DOI:** 10.3389/fphys.2021.665044

**Published:** 2021-11-08

**Authors:** Jacob T. Bonafiglia, Nicholas Preobrazenski, Brendon J. Gurd

**Affiliations:** ^1^School of Kinesiology and Health Studies, Queen’s University, Kingston, ON, Canada; ^2^Faculty of Medicine, University of Ottawa, Ottawa, ON, Canada

**Keywords:** individual response, interindividual variability, trainability, exercise training, responders, non-responder analysis

## Abstract

**Background:** Many reports describe statistical approaches for estimating interindividual differences in trainability and classifying individuals as “responders” or “non-responders.” The extent to which studies in the exercise training literature have adopted these statistical approaches remains unclear.

**Objectives:** This systematic review primarily sought to determine the extent to which studies in the exercise training literature have adopted sound statistical approaches for examining individual responses to exercise training. We also (1) investigated the existence of interindividual differences in trainability, and (2) tested the hypothesis that less conservative thresholds inflate response rates compared with thresholds that consider error and a smallest worthwhile change (SWC)/minimum clinically important difference (MCID).

**Methods:** We searched six databases: AMED, CINAHL, EMBASE, Medline, PubMed, and SportDiscus. Our search spanned the aerobic, resistance, and clinical or rehabilitation training literature. Studies were included if they used human participants, employed standardized and supervised exercise training, and either: (1) stated that their exercise training intervention resulted in heterogenous responses, (2) statistically estimated interindividual differences in trainability, and/or (3) classified individual responses. We calculated effect sizes (ES_IR_) to examine the presence of interindividual differences in trainability. We also compared response rates (*n* = 614) across classification approaches that considered neither, one of, or both errors and an SWC or MCID. We then sorted response rates from studies that also reported mean changes and response thresholds (*n* = 435 response rates) into four quartiles to confirm our ancillary hypothesis that larger mean changes produce larger response rates.

**Results:** Our search revealed 3,404 studies, and 149 were included in our systematic review. Few studies (*n* = 9) statistically estimated interindividual differences in trainability. The results from these few studies present a mixture of evidence for the presence of interindividual differences in trainability because several ES_IR_ values lay above, below, or crossed zero. Zero-based thresholds and larger mean changes significantly (both *p* < 0.01) inflated response rates.

**Conclusion:** Our findings provide evidence demonstrating why future studies should statistically estimate interindividual differences in trainability and consider error and an SWC or MCID when classifying individual responses to exercise training.

**Systematic Review Registration:** [website], identifier [registration number].

## Introduction

In 1999, the seminal Health, Risk Factors, Exercise Training, and Genetics (HERITAGE) Family Study reported individual cardiorespiratory fitness responses ranging from approximately −100 to +1,000 ml/min following 20 weeks of supervised and standardized aerobic exercise training (Bouchard et al., 1999). Early studies following HERITAGE continued to examine individual responses to exercise training by (1) interpreting a wide range of observed responses as evidence that exercise training causes interindividual variability and (2) classifying individuals as “responders” or “non-responders” if their observed response was above or below zero, respectively [reviewed in Williamson et al. (2017), Ross R. et al. (2019), Bonafiglia et al. (2020)]. In 2015, biostatisticians raised concerns with these approaches and have advocated for more rigorous statistical approaches when estimating interindividual variability and classifying individual responses (Atkinson and Batterham, 2015; Hecksteden et al., 2015; Hopkins, 2015). Despite many subsequent reviews echoing these concerns (reviews listed in Supplementary Table S1), we are aware of several studies published in the past year that did not adopt these statistical approaches (Marsh et al., 2020; Thomas et al., 2020; Vellers et al., 2020; Wang et al., 2020). Because no study has systematically reviewed the approaches used to examine individual responses, it is unclear whether these recent studies are representative of the exercise training literature.

Estimating interindividual variability requires partitioning the variability in outcome measurements caused by exercise training *per se*, herein referred to as interindividual differences in trainability, from the variability caused by random measurement error and within-subject variability (Bonafiglia et al., 2019a). Within-subject variability refers to real physiological responses resulting from changes in behavioral or environmental factors such as diet, sleep, and physical activity outside of a standardized exercise intervention (Bonafiglia et al., 2019a). In 2015, biostatisticians recommended statistical approaches that estimate interindividual differences in trainability by partitioning error or within-subject variability (Atkinson and Batterham, 2015; Hecksteden et al., 2015; Hopkins, 2015). Since then, many reviews have used data simulations or theoretical arguments to emphasize the importance of partitioning error or within-subject variability to encourage researchers to adopt these statistical approaches (Williamson et al., 2017; Swinton et al., 2018; Atkinson et al., 2019; Bonafiglia et al., 2019a; Ross R. et al., 2019; Voisin et al., 2019; Chrzanowski-Smith et al., 2020; Dankel and Loenneke, 2020). What remains unclear is whether the exercise training literature has adopted the recommended statistical approaches of biostatisticians. If the literature has not adopted these approaches, additional lines of evidence (i.e., beyond data simulations and theoretical arguments) may be required to persuade researchers to adopt a statistical approach that partitions error or within-subject variability when attempting to examine individual response heterogeneity.

With respect to classifying individual responses, labeling someone as a “non-responder” to exercise should be avoided because individuals can: (1) demonstrate individual patterns of response across a range of outcomes [e.g., the VO_2_max of an individual may “respond” positively while their body fat percentage may “not respond” (Barber et al., 2021)], (2) respond differently following different exercise doses (Bonafiglia et al., 2016; Montero and Lundby, 2017; Marsh et al., 2020), or (3) respond differently to repeated exposure to the same training intervention (Del Giudice et al., 2020). Further, given the difficulty in delineating changes caused by exercise *vs.* behavioral or environmental factors, “responders” and “non-responders” should not be interpreted as individuals who responded or did not respond to exercise *per se* (Swinton et al., 2018). Instead, “responders” and “non-responders” should be interpreted as individuals who did or did not experience benefit following the completion of an exercise training intervention (Swinton et al., 2018). To reduce the risk of misclassifying individuals who did not benefit as “responders,” many reports have recommended that “responders” should be classified as individuals whose observed change in a given outcome exceeds the smallest worthwhile change (SWC) or a minimum clinically important difference (MCID) after accounting for random measurement error (Bonafiglia et al., 2018, 2019b; Hecksteden et al., 2018; Swinton et al., 2018; Ross R. et al., 2019). In support of these recommendations, we (Schulhauser et al., 2020) and others (Hecksteden et al., 2018) found that thresholds not considering error and/or a SWC or MCID inflate “response rates” compared with more conservative thresholds that consider both error and a SWC or MCID. However, recent studies have classified “responders” and “non-responders” using non-conservative thresholds (Marsh et al., 2020; Thomas et al., 2020). Whether this observation is representative of the classification approaches used in the exercise training literature is unknown. Further, given that previous reports (Hecksteden et al., 2018; Schulhauser et al., 2020) included few outcomes (*n* = 6 and 1) and small sample sizes (*n* = 40 and 84), corroborating the inverse relationship between threshold conservativeness and response rates with a larger dataset may provide more compelling evidence to convince researchers to consider error and a SWC or MCID when classifying individual responses to exercise training.

The primary purpose of the present review was to determine the extent to which studies in the exercise training literature have adopted approaches to statistically estimate interindividual differences in trainability and consider error and an SWC or MCID when classifying individual responses. We performed a systematic review that spanned the aerobic, resistance, and clinical/rehabilitation training literature. We developed our search criteria to only include studies that examined individual responses (i.e., analyzed individual responses or commented on response heterogeneity), and our analyses, therefore, do not contain data from studies that did not consider individual responses.

## Materials and Methods

This systematic review followed the Preferred Reporting Items for Systematic Reviews and Meta-Analyses (PRISMA) checklist (see Supplementary Table S2). The study selection process was conducted using Covidence’s systematic review software (Veritas Health Innovation Ltd., Australia). Table 1 includes a list of definitions of individual response terms that we use throughout this review.

**TABLE 1:** Definitions of individual response terms used throughout the present review.

Term	Definition
***Terms related to interindividual differences in trainability***	
Random measurement error	Source of random variation caused by technical error and day-to-day biological variability
Within-subject variability	Source of random variation caused by real physiological changes that occur due to changes in behavioral and/or environmental factors external to the prescribed intervention
Interindividual differences in trainability	Variability in outcome measurements caused by exercise training; also referred to as “true response variability” or “the subject-by-training interaction” (Atkinson and Batterham, 2015; Hecksteden et al., 2015)
SD_IR_	A statistical estimate of interindividual differences in trainability calculated by subtracting observed variability in control from exercise groups
ES_IR_	An effect size of the SD_IR_ estimate
***Terms related to classifying individual response***	
“Responder”	An observed response that exceeds a given response threshold; importantly, this term should be applied in an outcome and training-specific manner (e.g., “VO_2_max responder to aerobic training”)
Response threshold	The threshold used to classify “responders”
Zero-based thresholds	Classification method that uses zero as the response threshold whereby “responders” have observed responses exceeding zero
Quantiles	An arbitrary classification approach that guarantees a fixed percentage of “responders” (e.g., using quartiles to identify the top 25% of participants as “responders”)
TE	Typical error; calculated to account for random measurement error when classifying individual responses (Hopkins, 2000)
MCID	Minimum clinically important difference; a non-arbitrary threshold for classifying individual responses based on evidence of clinically relevant changes [e.g., 1 MET improvements for cardiorespiratory fitness because this change is associated with risk reduction of all-cause mortality (Ross et al., 2016)]
SWC	Smallest worthwhile change; a response threshold calculated as 0.2 multiplied by baseline standard deviation and is recommended when an evidence-based MCID is not available
Response rate	The proportion of “responders” within a given group under specific classification parameters and training conditions

### Eligibility Criteria

Studies were included in the systematic review if they met all of the following inclusion criteria: (1) were an original, published research study (including novel re-analyses, which refer to secondary analyses of datasets where individual responses were not examined in primary reports), (2) used human participants, (3) employed a minimum of 2 weeks (or six sessions) of standardized and supervised exercise training, and (4) examined individual responses to exercise training by (i) stating that exercise training resulted in heterogeneous responses without using a statistical approach to estimate interindividual differences in trainability (e.g., commenting on response heterogeneity or a wide range of individual responses in their results, discussion, or conclusions); (ii) adopting a statistical approach to estimate interindividual differences in trainability (approaches listed in our Supplementary Table S3) (Atkinson and Batterham, 2015; Hecksteden et al., 2015, 2018; Hopkins, 2015; Dankel and Loenneke, 2020); and/or (iii) classifying individual responses. The latter criteria are not necessarily mutually exclusive. For example, Hecksteden et al. (2018) adopted the standard deviation of individual response (SD_IR_) to estimate interindividual differences in trainability (criteria ii) and classified individual responses (criteria iii). Conversely, Yu et al. (2020) adopted the SD_IR_ but did not classify (criteria ii only), and Bonafiglia et al. (2018) did not adopt the SD_IR_ but did classify (criteria iii only). Studies were excluded if they did not meet all of the inclusion criteria, or if the manuscript was not available (i.e., conference abstract only) or not in English.

### Literature Search and Study Selection

We conducted a literature search in AMED, CINAHL, EMBASE, Medline, PubMed, and SportDiscus on March 28, 2020. A second, identical up-to-date search took place on January 6, 2021. The search strategy incorporated two main concepts: exercise training and individual response. A complete list of synonyms or related terms for these two main concepts was combined with “OR” (see Supplementary Table S4 for full list), and the search strategy combined the two separate synonym lists with “AND.” Titles and abstracts were extracted from the database searches, and Covidence automatically removed duplicates.

Study selection followed a two-step process and was independently completed by JTB and NP. BJG resolved disagreements (*n* = 2 total). First, titles and abstracts were screened to identify studies that appeared to meet eligibility criteria. Second, full texts were downloaded for articles that passed title and abstract screening to determine their eligibility. Studies removed during full-text screening were assigned a reason for exclusion. Final analyses included studies that passed both levels of study selection. We used the Cochrane Collaboration Risk of Bias Assessment Tool (Higgins et al., 2011) to assess the risk of bias. We reviewed the protocol and/or primary publications to evaluate the risk of bias for studies that included re-analyses of previously published data.

### Data Extraction

JTB and NP performed data extraction using a predetermined data collection template, which included the variables provided in our Supplementary Table S5 along with response rates, mean changes, and response thresholds. These two reviewers met to compare extracted data and resolve discrepancies. JTB and NP dichotomously categorized all included studies based on whether they did or did report using a statistical approach (Atkinson and Batterham, 2015; Hecksteden et al., 2015, 2018; Hopkins, 2015; Dankel and Loenneke, 2020) to estimate interindividual differences in trainability. Because adopting a statistical approach is needed to partition the sources of variation, this dichotomous categorization allowed us to determine how many studies may have overlooked the confounding influence of random measurement error and within-subject variability on response variability. This dichotomous categorization is further warranted because studies not adopting a statistical approach, and thus not accounting for the confounding sources of variation, risk erroneously interpreting variability in observed responses as evidence of interindividual differences in trainability. After this initial categorization, JTB and NP then sorted studies that classified individual responses into three categories based on whether they considered an error and/or an SWC or MCID (see Table 2 for details).

**TABLE 2:** Categorization details for the primary analysis.

Category	Description
***Did studies use a statistical approach to estimate interindividual differences in trainability?***	
Yes	**Used a statistical approach** (Atkinson and Batterham, 2015; Hecksteden et al., 2015, 2018; Hopkins, 2015; Bonafiglia et al., 2019a) that accounts for random measurement error or within-subject variability to estimate interindividual differences in trainability
No	This category included all studies that commented on variability (e.g., “we observed a wide range of individual responses”) or classified individual responses **without performing statistical analysis** to estimate interindividual differences in trainability
***How did studies classify individual responses?***	
Did not consider error or a SWC or MCID	Classified “responders” and “non-responders” using quantiles or zero-based thresholds (e.g., “responders” identified as individuals with observed responses exceeding zero). These approaches **do not** consider error or a SWC or MCID
Considered error or a SWC or MCID^*^	Used a response threshold that **either** considered error **or** a SWC or MCID
Considered error and a MCID/SWC	Used a response threshold that considered **both** error and a SWC or MCID

*Studies that only considered error were combined with studies that only considered an SWC or MCID based on our observation that these approaches generally result in similar response thresholds (e.g., Johannsen et al., 2013; Hecksteden et al., 2018; Bonafiglia et al., 2019b; Schulhauser et al., 2020). Bold indicates to visually emphasize and differentiate descriptions of categories.

We extracted basic study characteristics such as training protocols, participant characteristics, and measured outcomes from all included studies (see Supplementary Table S5 for study characteristics). For studies that statistically estimated interindividual differences in trainability, we extracted sample sizes and standard deviations (SD) of baseline values and change scores. We used WebPlotDigitizer (Rohatgi, 2018) to calculate SDs of change scores from two studies (Steele et al., 2017; Hammond et al., 2019) that did not report these values but presented individual data in their figures. For studies that classified individual responses, we extracted mean changes, thresholds used to classify “responders,” and response rates. For the purposes of this review, response rate refers to the proportion of “responders” for a given outcome following a specific exercise or control condition (Table 1).

### Data Analysis

In addition to our primary analysis, we conducted two analyses to demonstrate limitations with not statistically estimating interindividual differences in trainability or considering error and an SWC or MCID. First, we calculated estimates of interindividual differences in trainability from included studies that have adopted a statistical approach (Supplementary Table S3) to determine how many outcomes provided evidence of variability caused by exercise training *per se*. Second, we examined the impact of threshold conservativeness on response rates. Details for these two analyses are provided in the following sections.

#### Interindividual Differences in Trainability

For studies that statistically estimated interindividual differences in trainability, we calculated the SD_IR_, a statistic that estimates the presence of interindividual differences in trainability (see Bonafiglia et al., 2019a for a detailed explanation), for each outcome using the following equation (Atkinson and Batterham, 2015; Hopkins, 2015):

(1) $$S\,D_{I\,R}=\sqrt{S\,D_{E\,X}^{2}-S\,D_{C\,T\,R\,L}^{2}}$$

where SD_EX_ and SD_CTRL_ represent the SD of change scores from the exercise (EX) and control group (CTRL), respectively. Positive SD_IR_ values (i.e., SD_EX_ < SD_CTRL_) suggest that interindividual differences in trainability exist. An SD_IR_ cannot be calculated if SD_CTRL_ exceeds SD_EX_ because you cannot take the square root of a negative number (Eq. 1). In these instances, researchers can either report not being able to calculate an SD_IR_, or they can switch SD_CTRL_ with SD_EX_ in Eq. 1 and report the value as a negative SD_IR_. Both incalculable and negative SD_IR_ values should be interpreted as a lack of evidence for interindividual differences in trainability (Bonafiglia et al., 2019a). For studies that had multiple exercise groups, we amalgamated the data of the exercise groups according to chapter 7.7.3.8 in the Cochrane Handbook (Higgins et al., 2019) to calculate one SD_EX_ value and thus one SD_IR_ value for each outcome in each study. The standard error (SE) for each SD_IR_ value was calculated to construct 90% confidence intervals (CIs) using the following equations (Hopkins, 2015; Hecksteden et al., 2018):

(2) $$S\,E=\sqrt{2\,\left(\frac{S\,D_{E\,X}^{4}}{\left(n_{E\,X}-1\right)}+\frac{S\,D_{C\,T\,R\,L}^{4}}{\left(n_{C\,T\,R\,L}-1\right)}\right)}$$

(3) $$90\%\,C\,I\,L\,i\,m\,i\,t\,s=\sqrt{S\,D_{I\,R}^{2}\pm 1.65\times S\,E}$$

where *n*_EX_ and *n*_CTRL_ represent sample sizes for the EX and CTRL groups, respectively. To visually present these data from different outcomes in one figure, we standardized SD_IR_ values by calculating unitless effect sizes (denoted as ES_IR_) using the following equation (Hopkins, 2015):

(4) $$E\,S_{I\,R}=\frac{S\,D_{I\,R}}{S\,D_{B\,S\,L.P\,o\,o\,l\,e\,d}}$$

where SD_BSL.Pooled_ represents the pooled SD of baseline values from the EX and CTRL groups. Upper and lower CI limits were also divided by SD_BSL.Pooled_ to construct 90% CIs for ES_IR_ values (Hopkins, 2015).

The interpretation of interindividual differences in trainability for each outcome was based on the positions of 90% CIs: CIs laying fully above zero suggested that interindividual differences in trainability were present, whereas CIs crossing or laying fully below zero indicated a lack of evidence for interindividual differences in trainability.

#### Analysis of Variances on Response Rates

We performed one-way analysis of variance (ANOVA) comparing response rates across the three response classification categories outlined in Table 2. We performed an ANOVA that compared response rates across every outcome and another ANOVA on the most commonly reported outcome. The ANOVA on the most commonly reported outcome was performed to determine whether the variance introduced by comparing response rates across different outcomes impacted our ability to detect significance. We excluded studies that used quantiles because these approaches guarantee a fixed percentage of responders or non-responders and therefore do not provide estimates of response rates. To determine whether standardized mean changes impacted our ANOVA on all outcomes, we also performed an ANCOVA with a standardized mean ($\bar{x}$) changes inputted as a covariate. Specifically, we first extracted available pre and post-training means and SDs for each outcome to calculate Cohen’s *d* values using the following equation:

(5) $$d_{a\,v}=\frac{{\bar{x}}_{P\,O\,S\,T}-{\bar{x}}_{P\,R\,E}}{P\,o\,o\,l\,e\,d\,S\,D}$$

where *d*_av_ refers to a Cohen’s *d* value for a within-subject standardized mean change (Lakens, 2013), and pooled SD was calculated as the standard deviation of pre values plus the standard deviation of post values divided by two. Because many (130 out of 298) of the sample sizes included in our ANCOVA were less than 20, we then converted *d*_av_ values to Hedge’s *g* values (*g*_av_) using the following equation:

(6) $${\text{Hedge}}^{{}^{\prime }}\,\text{s}\,g_{a\,v}=d_{a\,v}\times \left(1-\frac{3}{4\times \left(n_{P\,O\,S\,T}+n_{P\,R\,E}\right)-9}\right)$$

Additionally, our ancillary hypothesis investigated whether response rates were a function of mean change relative to the response threshold. We based this hypothesis on findings from two of our recent studies: (1) larger mean changes produce larger response rates at a given response threshold (Bonafiglia et al., 2021b), and (2) smaller response thresholds produce larger response rates at a given mean change (Schulhauser et al., 2020). We tested this hypothesis using outcomes with reported response rates, mean changes, and response thresholds; outcomes could not be included if any of these three parameters were not reported. Data from both exercise and non-exercising control groups were included in this analysis. Our dependent variable was response rates (in percentage), and our independent variable was calculated as the mean change divided by the response threshold. This approach standardized our independent variable and thus allowed us to compare response rates across different outcomes. We could not include response rates from studies using zero-based thresholds because mean changes cannot be divided by a response threshold of zero. We then sorted outcomes into four response rate quartiles: response rates less than or equal to (1) 100%, (2) 75%, (3) 50%, or (4) 25%. Following this, we used a one-way ANOVA to compare standardized mean changes across these response rate quartiles. We identified outliers within each quartile as *z*-scores that were either >2.58 or <−2.58, and we removed these data before running our ANOVA.

For all ANOVAs, significant main effects were followed by Bonferroni *post hoc* tests. These statistical tests were performed on GraphPad Prism version 9 with the ANCOVA performed on SPSS version 26. All data are presented as means ± standard deviation.

## Results

### Study Selection

**FIGURE 1:**
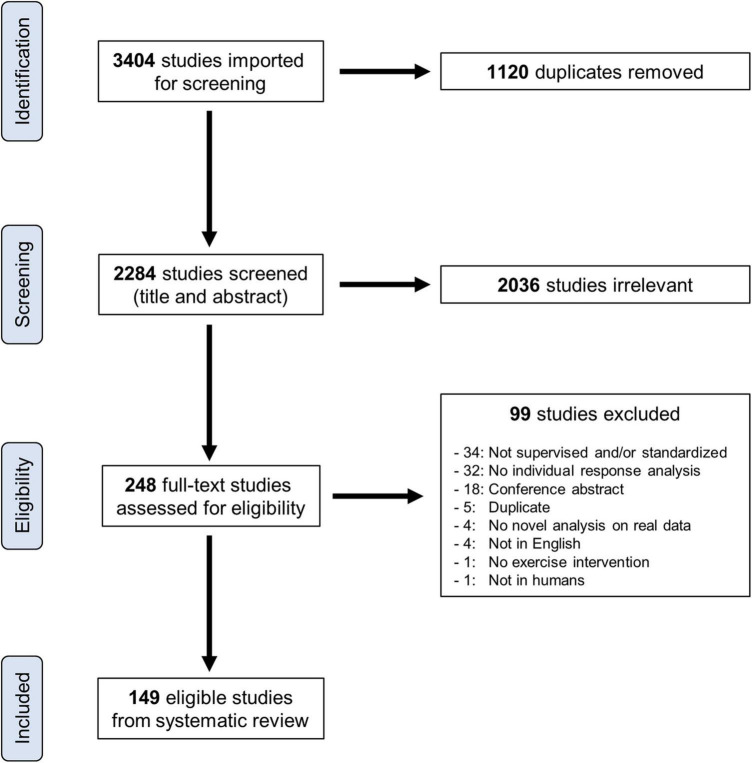
Flow diagram of the study selection process.

Figure 1 presents a flow diagram of the study selection process. The literature search retrieved 3,404 studies, and Covidence removed 1,120 duplicates. Two thousand two hundred eighty-four studies entered title and abstract screening, and 2,036 were deemed irrelevant and were subsequently excluded. Full texts were then downloaded for 248 studies, and 99 were excluded (reasons provided in Figure 1), leaving a total of 149 included studies. Study details, measured outcomes, analytical approaches, and participant characteristics for these 149 studies can be found in the Supplementary Table S5. Most included studies had an unclear-high risk of bias (results presented in Supplementary Table S6). For ease of viewing, in-text references for all 149 included studies are provided at the end of this manuscript (see Appendix). Table 3 presents study characteristics and analysis categories (categories outlined in Table 2) for these 149 studies.

**TABLE 3:** Overview of study details for the 149 studies included in our systematic review.

Type of training	# of studies (% all studies included)	Avg. duration in weeks (SD)	Avg. frequency (SD)	# in males only	# in females only	# in both sexes	Variability categories^a^		Response classification categories^b^		
							# “Yes”	# “No”	# No TE or SWC or MCID	# TE or SWC or MCID	# TE and SWC or MCID
**Aerobic**	74 (50%)	13.1 (10.1)	3.9 (1.7)	16 (22%)	9 (12%)	48^c^ (65%)	68 (92%)	6 (8%)	14 (27%)	30 (58%)	8 (15%)
**Resistance**	32 (22%)	14.3 (9.3)	2.7 (0.8)	13 (41%)	8 (25%)	11 (34%)	31 (97%)	1 (3%)	12 (44%)	15 (56%)	0 (0%)
**Aerobic and Resistance**	35 (23%)	17.1 (8.9)	3.3 (0.7)	4 (11%)	5 (14%)	26 (74%)	33 (94%)	2 (6%)	10 (37%)	16 (59%)	1 (2%)
**Other**	8 (5%)	11.4 (6.2)	4.1 (2.9)	1 (13%)	0 (0%)	7 (87%)	8 (100%)	0 (0%)	1 (14%)	5 (72%)	1 (14%)

*^*a*^Variability categories outlined in Table 2 (“Did studies use a statistical approach to estimate interindividual differences in trainability?”); ^*b*^Classification categories outlined in Table 2 (“How did studies classify individual responses?”); ^*c*^One group did not provide sex composition. Note that percentages for the variability categories are based on the total number of studies within each type of training (aerobic = 74; resistance = 32; aerobic and resistance = 35; other = 8), whereas the percentages for response classification categories are based on the number of studies that classified individual responses within each type of training (aerobic = 52; resistance = 27; aerobic and resistance = 27; other = 7).*

### Timeline of Studies Examining Individual Responses to Exercise Training

**FIGURE 2:**
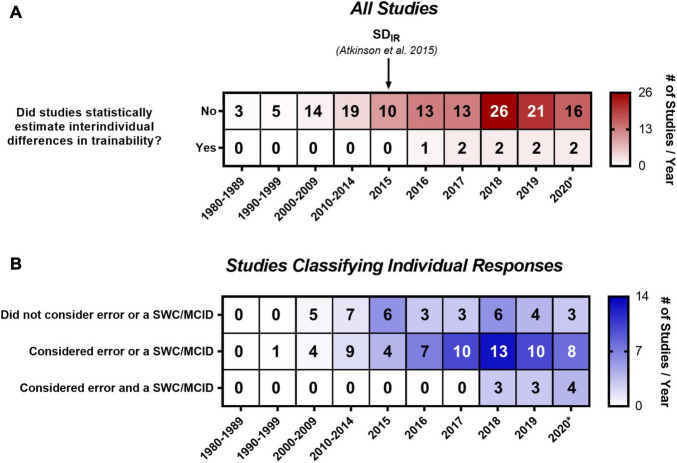
Timeline and heatmap of the 149 studies included in our systematic review. Numbers refer to a total number of studies in each cell, whereas shading depicts the total number of studies divided by the number of years included in that column. **(A)** Includes all studies sorted by whether studies used a statistical approach to estimate interindividual differences in trainability, and **(B)** includes the 116 studies that classified individual responses sorted by the classification categories outlined in Table 2. 2020* includes studies published in 2020 and up to the updated literature search (January 6, 2021). MCID, minimum clinically important difference; SD_IR_, the standard deviation of individual responses; SWC, smallest worthwhile change. **(B)** Only contains 113 of the 116 studies that classified individual responses because three studies did not report how individuals were classified (Hubal et al., 2005; Hagstrom and Denham, 2018; Peltonen et al., 2018).

We created two timelines of the studies included in our analysis (Figure 2). Figure 2A includes all studies and is sorted based on whether studies used a statistical approach to estimate interindividual differences in trainability. The number of studies examining individual responses to exercise training has increased substantially since 1999, when the findings from the HERITAGE Family Study were published, with the majority (72.5%; 108/149) being published within the last 5 years (i.e., 2015 onward). The paper by Atkinson and Batterham (2015) is highlighted in Figure 2A because it describes the SD_IR_ approach and, to our knowledge, is the first article in the exercise literature outlining how to statistically estimate the presence of interindividual differences in trainability. It was therefore unsurprising that no study before 2015 statistically estimated interindividual differences in trainability (Figure 2A). However, strikingly few studies (∼9.5%; 8/84 studies) published in 2017 onward [i.e., after Atkinson et al.’s (2019) SD_IR_ paper] statistically estimated interindividual differences in trainability. Seven of these studies (Steele et al., 2017; Williamson et al., 2017, 2018; Bonafiglia et al., 2019a; Hammond et al., 2019; Walsh et al., 2020; Yu et al., 2020) used the SD_IR_, and one study explored different SD_IR_ approaches using a variety of statistical parameters (Hecksteden et al., 2018). The 2016 study by Leifer et al. (2016) used Levene’s tests to compare variability in observed responses between control and exercise groups – an approach that follows the same principles as the SD_IR_ (Bonafiglia et al., 2019a).

A percentage of 77.9% (116/149) of all included studies classified individual responses, and a timeline of these studies sorted by the three categories outlined in Table 2 is presented in Figure 2B. 31.9% of studies (37/116) classified individuals using an approach that did not consider error or an SWC or MCID (e.g., zero-based thresholds or quantiles). 56.9% of studies (66/116) considered error (66.7%; 44/66) or an SWC or MCID (33.3%; 22/66), and the most common approach in this category (37.9%; 25/66) was classifying responders as individuals whose observed response exceeded a threshold of two times the typical error. Only 8.6% of studies (10/116) considered both error and an SWC or MCID. The most common approach (90%; 9/10) in this last category was classifying responders as individuals with confidence intervals, built using the typical error of measurement and constructed around observed responses, that lay fully above an SWC or MCID. Supplementary Table S5 provides more information on the specific classification approaches used in each study. Three studies (Hubal et al., 2005; Hagstrom and Denham, 2018; Peltonen et al., 2018) were not categorized because they did not report enough information on how they classified individual responses.

### Studies Adopting Approaches for Estimating Interindividual Differences in Trainability

We were unable to calculate ES_IR_ values from three of the nine studies that statically estimated interindividual differences in trainability because they did not report SD of baseline measures and/or change scores (Williamson et al., 2017, 2018; Bonafiglia et al., 2019a). All three of these studies included novel re-analyses of previously published data. Williamson et al. (2017) reported greater variability in VO_2_max responses in a control group compared with an exercise group, the 2018 meta-analysis of Williamson et al. (2018) demonstrated trivial interindividual differences in trainability for body weight, and we reported moderate-large variability in behavioral factors (e.g., dietary habits and sedentary time) following a controlled exercise intervention (Bonafiglia et al., 2019a).

**FIGURE 3:**
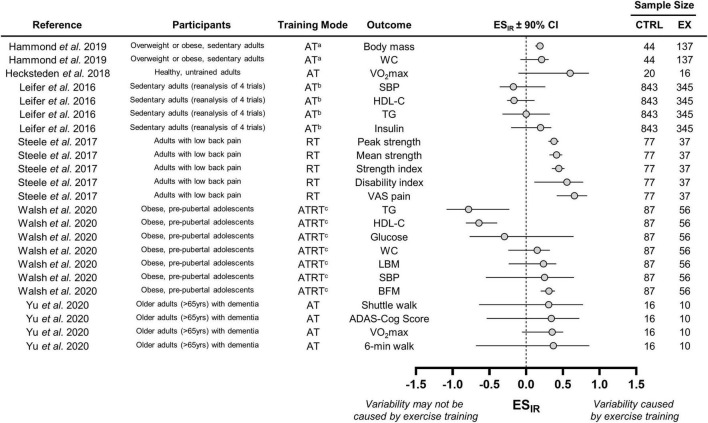
Forest plot of the studies that statistically estimated interindividual differences in trainability. CTRL, control group; EX, exercise training group; WC, waist circumference; VO_2_max; maximal oxygen uptake; SBP, systolic blood pressure; HDL-C, high-density lipoprotein cholesterol; TG, triglyceride; VAS, visual analog scale; LBM, lean body mass; BFM, body fat mass; ADAS-cog, Alzheimer’s disease assessment scale-cognitive subscale. ^a^We combined data from three aerobic training groups (see section “Interindividual Differences in Trainability”); ^b^Data from four trials combined by Leifer et al. (2016); ^c^We combined data from the three training groups (see section “Interindividual Differences in Trainability”), which included an aerobic, resistance, and combined training group.

We calculated ES_IR_ values in the remaining six studies. These values with 90% CIs along with basic details regarding participant characteristics and training modes are presented in Figure 3. Combining data across the three aerobic training groups from Hammond et al. (2019) resulted in positive ES_IR_ values for body mass and waist circumference. However, we can only conclude that interindividual differences in trainability were present for body mass because the 90% CI for waist circumference crossed zero. The aerobic, resistance, and aerobic plus resistance groups were also combined in the study Walsh et al. (2020). Responses in body composition and cardiometabolic health from Walsh et al. (2020) revealed mixed evidence of interindividual differences in trainability because ES_IR_ 90% CIs lay above, below, or crossed zero. ES_IR_ estimates from the Leifer et al. (2016). The Leifer et al. (2016) study also presented a mixture of 90% CI positions for indices of cardiometabolic health. The ES_IR_ values from Hecksteden et al. (2018) and Yu et al. (2020), who measured fitness parameters following aerobic training, were all positive but had large 90% CIs that crossed zero. These large CIs were likely attributable to the small sample sizes of these two studies. Interestingly, only the study by Steele et al. (2017), which examined strength responses to resistance training, revealed consistent evidence of interindividual differences in trainability as all ES_IR_ 90% CIs lay above zero.

### Analysis of Variances on Response Rates

**FIGURE 4:**
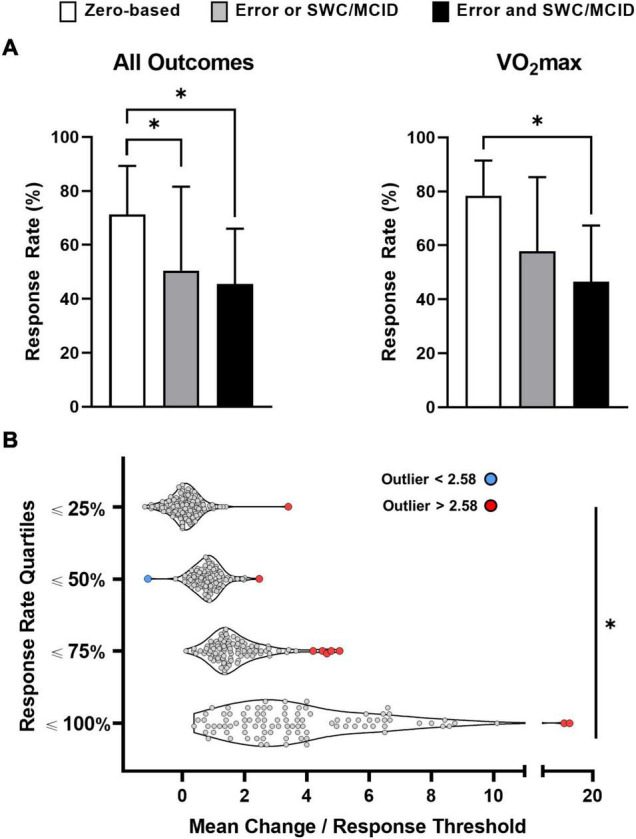
Impact of classification approach [**A**; *n* = 614 (all outcomes); *n* = 75 (maximal oxygen uptake, VO_2_max)] and mean change **(B)** on response rates. Table 2 outlines the classification categories outlined in **(A)**. *Significant at *p* < 0.01.

We obtained response rates for 614 outcomes from the 116 studies that classified individual responses. 71, 491, and 52 response rates were obtained from zero-based thresholds, approaches that considered error or an SWC or MCID, or both error and an SWC or MCID, respectively. Our one-way ANOVA with all 614 response rates (Figure 4A; left panel) was significant (*p* < 0.01) with zero-based thresholds producing a significantly (*p* < 0.01) higher mean response rate (71.22 ± 18.09%) compared with approaches that considered one of (50.53 ± 31.08%) or both error and an SWC or MCID (45.49 ± 20.52%). Our second ANOVA was performed on VO_2_max because this outcome had the most response rates (*n* = 75) compared with other outcomes (next three outcomes with the most response rates: various strength measures, *n* = 51; waist circumference, *n* = 20; body weight, *n* = 17). The one-way ANOVA on VO_2_max response rates (Figure 4A; right panel) was also significant (*p* < 0.05) with zero-based thresholds resulting in a significantly (*p* < 0.05) higher mean response rate (78.42 ± 12.98%) compared with approaches that considered both error and an SWC or MCID (46.50 ± 20.85%). We extracted 298 Hedge’s *g* values, and our ANCOVA and associated *post hoc* tests remained significant indicating that standardized mean changes did not confound the relationship between classification category and response rate (Figure 4A).

Response rates, mean changes, and response thresholds were reported for 435 of the 614 outcomes. Our ancillary analysis (Figure 4B) on these 435 outcomes revealed significant differences in standardized mean changes (mean change divided by response threshold) across each quartile: ≤25% = 0.09 ± 0.46; ≤50% = 0.83 ± 0.41; ≤75% = 1.58 ± 0.69; ≤100% = 3.62 ± 2.19 (ANOVA and all *post hoc p* values < 0.01). We identified ten outlying data points, and only one lay below the mean (blue data point; all other outliers in red; Figure 4B). The two outliers in the upper quartile (response rates ≤100%) had very large standardized mean changes, and both data points represent strength gains (leg press one-repetition maximum) from two resistance training groups in the same study (Barbalho et al., 2017). The remaining eight outlying data points came from six studies (Alvarez et al., 2017b,c; Álvarez et al., 2018a,d; Delgado-Floody et al., 2019; Ramírez-Vélez et al., 2019) produced by the same research group, and homeostasis model assessment-estimated insulin resistance measurements accounted for four of these outliers.

## Discussion

This is the first systematic review to investigate the approaches used to examine individual responses to exercise training. Our search revealed a large number of eligible studies (*n* = 149; Figure 1) that spanned the aerobic, resistance, and clinical/rehabilitation training literature. Our primary analysis revealed that few studies have statistically estimated whether exercise training causes interindividual differences in trainability (Figure 2A). This finding indicates that the majority of studies may have inappropriately interpreted variability in observed responses as evidence of interindividual differences in trainability. In support of this speculation, our review highlighted several ES_IR_ values that either fell below zero or had a 90% CI crossing zero (Figure 3). Given that many recent studies have not adopted the statistical approaches described in previous reviews (listed in Supplementary Table S3), we hope our findings help persuade researchers to adopt these approaches when estimating the existence of interindividual differences in trainability in the future work.

Our additional analyses found that few studies considered an error and an SWC or MCID when classifying individual responses (Figure 2B). Our analyses on response rates confirmed the hypotheses that: (1) thresholds not considering error and an SWC or MCID inflate response rates (Figure 4A), and (2) larger mean changes produce larger response rates (Figure 4B). Given the disconnect between the many reviews (listed in Supplementary Table S1), highlighting the importance of considering error and an SWC or MCID and the few studies doing so (Figure 2B), our findings hopefully encourage researchers to consider error and an SWC or MCID when classifying individual responses in future studies.

### Interindividual Differences in Trainability

Our ES_IR_ calculations highlighted mixed evidence for the presence of interindividual differences in trainability (Figure 3). For instance, resistance training in the Steele et al. (2017) study appeared to cause variability in strength responses, whereas aerobic training in the Leifer et al. (2016) re-analysis may not have led to interindividual differences in cardiometabolic responses because the 90% CIs crossed zero. Interestingly, fasting insulin from the Leifer et al. (2016) study and peak strength from the Steele et al. (2017) study had similar ES_IR_ values, but only peak strength revealed evidence of interindividual differences in trainability because its 90% CI lay fully above zero (Figure 3). Because fasting insulin had a much larger sample size than peak strength (*n* = 1188 vs. *n* = 114), its larger 90% CI may reflect how blood-based physiological outcomes have larger random measurement error compared with strength-based performance outcomes. This observation may also highlight how random measurement error for a given outcome influences interpretations of interindividual differences in trainability. Future work should therefore avoid pooling data across different outcomes when estimating interindividual differences in trainability. Moreover, these discrepancies highlight how reading one of these studies in isolation may lead to either the conclusion that interindividual differences in trainability exist or do not exist (Leifer et al., 2016; Steele et al., 2017). Figure 3 presents ES_IR_ values across a range of outcomes and studies, highlighting how the existence of interindividual differences in trainability should be interpreted on an outcome, population, and study-specific basis.

The observation that most ES_IR_ values (18/25) lay below or crossed zero (Figure 3) adds to a growing body of literature questioning the presence of interindividual differences in trainability following standardized exercise training (Williamson et al., 2017, 2018; Del Giudice et al., 2020; Kelley et al., 2020, 2021; Bonafiglia et al., 2021a; Islam et al., 2021). For example, we recently reported wide-ranging changes in energy intake, diet composition, and sedentary time following a controlled exercise intervention (Bonafiglia et al., 2019a). This apparent variability in behavioral changes suggests that within-subject variability contributes substantially to the variability in observed responses. Recent studies have also demonstrated that individual cardiorespiratory fitness and skeletal muscle responses appear non-reproducible following repeated exposure to an identical exercise training intervention (Lindholm et al., 2016; Del Giudice et al., 2020; Islam et al., 2021). Assuming trainability of an individual is a stable and reproducible trait, this non-reproducibility provides further evidence suggesting that within-subject variability largely comprises the variability in observed responses (Hecksteden et al., 2015). Taken together, these findings indicate that it may be erroneous to assume that variability in observed responses reflects interindividual differences in trainability. Despite this indication, also highlighted in many previous reviews (listed in Supplementary Table S1), the majority of recent studies have not adopted a statistical approach to investigate the existence of interindividual differences in trainability (Figure 2A). Future work should therefore use a statistical approach to determine whether interindividual differences in trainability are present following exercise training (Atkinson and Batterham, 2015; Hecksteden et al., 2015, 2018; Hopkins, 2015; Dankel and Loenneke, 2020).

Although we focused on interindividual differences in trainability, other disciplines have reported a similar lack of evidence for “true response variability” following non-exercise interventions (Mills et al., 2020). For example, a recent meta-analysis of psychiatric assessment responses in patients with schizophrenia reported greater variability in control groups compared with treatment groups (Winkelbeiner et al., 2019). Rather than using the SD_IR_, these non-exercise studies utilized the “variability ratio” (Winkelbeiner et al., 2019; Mills et al., 2020), an approach that is similar to the SD_IR_ because it relies on the assumption of independence to estimate true response variability. In this context, the assumption of independence refers to the assumption that random measurement error and within-subject variability are equal between groups in a randomized controlled trial (Bonafiglia et al., 2019a). However, the variability ratio divides, instead of subtracts, the variability of observed responses in the treatment group by the variability of observed responses in the control group. The variability ratio was used in recent meta-analyses (reviewed in Mills et al., 2020) across scientific disciplines other than exercise and sport science to empirically test for the existence of “true response variability.”

### Classifying Individual Responses

We found that zero-based thresholds inflate response rates compared with classification approaches that consider error and an SWC or MCID (Figure 4A). These findings add to previous results derived from fewer outcomes and smaller datasets demonstrating that non-conservative thresholds increase the proportions of participants classified as “responders” (Hecksteden et al., 2018; Schulhauser et al., 2020). We hope our large dataset (i.e., 614 response rates from 116 studies) demonstrating inflated response rates with zero-based thresholds (Figure 4A) better convinces researchers to consider error and an SWC or MCID when classifying individual responses. Although considering error and an SWC or MCID will help conservatively identify individuals who experienced meaningful benefit, it is also possible these conservative thresholds increase the risk of failing to classify individuals who responded to exercise *per se* as “responders” (i.e., type II error). Conversely, less conservative thresholds (e.g., zero-based or considering one of error or an SWC or MCID only) increase the risk of classifying individuals who did not experience meaningful benefit as “responders” (i.e., type I error). While researchers can choose which risk of misclassification is more acceptable for their study, avoiding dichotomous classification and considering uncertainty (e.g., individual confidence intervals) can help reduce the risk of misclassifying “responders” or “non-responders” (Bonafiglia et al., 2018; Swinton et al., 2018).

Controlling for differences in response thresholds supported our ancillary hypothesis that larger mean changes produce larger response rates (Figure 4B). This finding corroborates our recent demonstration that larger mean changes explain why higher doses of exercise produce larger cardiorespiratory fitness response rates (Bonafiglia et al., 2021b). We speculate that the red outliers in the 25, 50, and 75% quartiles had large variability (or a single outlying data point), which resulted in a low response rate despite having a large standardized mean change (and *vice versa* for the blue data point in Figure 4B). However, analyzing these raw data is needed to confirm this speculation. Both analyses demonstrate how response rates are group statistics that highly depend on response thresholds (Figure 4A) and mean changes (Figure 4B; Atkinson et al., 2019). Future research should therefore avoid attributing lower response rates to reduced interindividual variability, an interpretation made in several studies included in our review (Wolpern et al., 2015; Dalleck et al., 2016; Byrd et al., 2019), and should recognize that response rates provide little if any, useful information at the individual level (Atkinson et al., 2019).

Although our results demonstrate *how* we should classify observed changes following exercise training (i.e., consider error and an SWC or MCID; Figure 4A), what remains less clear is *why* we should classify individuals in the first place. Although individual classification may help guide exercise prescription decision-making in clinical and applied settings, to our knowledge, only one study (Montero and Lundby, 2017) has utilized initial response classifications to guide subsequent exercise prescription decisions. A key challenge to individualizing exercise prescription is choosing a response threshold that incorporates an equipment-specific error estimate (Weatherwax et al., 2018a) and a change linked to clinical benefit (i.e., MCID). While equipment-specific errors can be easily generated using simple test re-test experiments, MCIDs are not available for many outcomes (Hopkins, 2000). For VO_2_max, we recently used an MCID of 1.0 MET because this change confers a ∼8–14% risk reduction in all-cause morbidity and mortality (Dorn et al., 1999; Bonafiglia et al., 2019b). When MCIDs are unavailable, researchers can use SWCs (i.e., 20% of baseline standard deviation) to estimate a threshold representing a small effect size (Swinton et al., 2018). It is important to emphasize that, unlike MCIDs, SWCs are clinically arbitrary and should not be used to gauge whether an individual has clinically benefited following exercise training. Future work is needed to examine the validity of the classification of individual responses for the purpose of optimizing exercise prescriptions and for establishing MCIDs for a wider range of outcomes.

### Limitations and Future Directions

The few studies that statistically estimated the presence of interindividual differences in trainability varied substantially in participant characteristics, training modes, and outcomes assessed (Figure 3). Recent meta-analyses have reported pooled SD_IR_ estimates indicating a lack of interindividual differences in trainability in body weight and body composition (Williamson et al., 2018; Kelley et al., 2020, 2021). Unlike the present study, these meta-analyses focused on a single outcome and used search criteria that identified all studies reporting standard deviations of change scores and not just those evaluating individual responses as we did in the present review. We did not pool ES_IR_ values because of the heterogeneity in populations (e.g., overweight adolescents, adults with low back pain, or older adults with cognitive impairment) and outcomes assessed (e.g., VO_2_max, perceptions of back pain, or an Alzheimer’s cognition score) across studies (Figure 3). Nevertheless, the observation that most ES_IR_ values (18/25) lay below or crossed zero supports conclusions from pooled estimates of single outcomes demonstrating a lack of interindividual differences in trainability. More studies should adopt the SD_IR_ approach to allow for additional population- and outcome-specific meta-analyses on other clinically relevant outcomes (e.g., VO_2_max, waist circumference, grip strength, etc.). Importantly, these meta-analyses can provide robust assessments of the presence or absence of interindividual differences in trainability.

Large variation in our dataset may also explain why response rates were not significantly different between studies that considered error or SWC or MCID *vs.* studies that considered both (Figure 4A). Comparing response rates across different thresholds within the same group (and thus mean change) overcomes this limitation by eliminating between-study variation in participant characteristics, training modes, and random measurement error. Although we and others have explored the relationship between response rates and thresholds using VO_2_max and exercise performance data (Hecksteden et al., 2018; Schulhauser et al., 2020), future work should confirm that failing to account for error and an SWC or MCID inflates response rates in different outcomes.

## Conclusion

The present systematic review found that despite many previous reviews (listed in Supplementary Table S1) advocating for more statistically sound approaches when examining individual responses, few studies have statistically estimated interindividual differences in trainability or used response classification thresholds that consider error and an SWC or MCID. We also presented ES_IR_ values that question the presence of interindividual differences in trainability, which demonstrates why it is inappropriate and potentially erroneous to assume variability in observed responses reflects interindividual differences in trainability. Further, we found that zero-based thresholds inflate response rates, which demonstrates why it is important to classify responses using an approach that considers both error and an SWC or MCID. Additionally, our analysis examining mean changes supported the notion that response rates are group statistics that provide little information about an individual’s response to exercise training. We hope our findings and novel data presentations better convince researchers to statistically estimate interindividual differences in trainability and consider error and an SWC or MCID in future work.

## Data Availability Statement

The original contributions presented in the study are included in the article/Supplementary Material, further inquiries can be directed to the corresponding author.

## Author Contributions

JTB and NP wrote the first draft of the manuscript. All authors contributed to conception and design of the study, conducted the systematic review, statistical analysis, and manuscript revision, read, and approved the submitted version.

## Conflict of Interest

The authors declare that the research was conducted in the absence of any commercial or financial relationships that could be construed as a potential conflict of interest.

## Publisher’s Note

All claims expressed in this article are solely those of the authors and do not necessarily represent those of their affiliated organizations, or those of the publisher, the editors and the reviewers. Any product that may be evaluated in this article, or claim that may be made by its manufacturer, is not guaranteed or endorsed by the publisher.

## Appendix

### In-Text Citations for the 149 Included Studies

*References A-I:*

(Bouchard et al., 1990, 1994, 1990, 2012; Dionne et al., 1991; Barbeau et al., 2003; Boule et al., 2005; Hubal et al., 2005; Hautala et al., 2006; Gross et al., 2007; Caudwell et al., 2009; Erskine et al., 2010; Davidsen et al., 2011; Croymans et al., 2013; Astorino and Schubert, 2014; Goddard et al., 2014; Chmelo et al., 2015; Churchward-Venne et al., 2015; Dalleck et al., 2015, 2016; Ahtiainen et al., 2016, 2020; Bonafiglia et al., 2016, 2018, 2021a,b; Garcia et al., 2016; Gurd et al., 2016; Hauser et al., 2016; Alvarez et al., 2017a,b,c; Barbalho et al., 2017; Chen et al., 2017; de Lannoy et al., 2017; Hvid et al., 2017; Astorino et al., 2018; Álvarez et al., 2018a,b,c,d, 2019; Bakker et al., 2018; Cadore et al., 2018; Díaz-Vegas et al., 2018; Edgett et al., 2018; Gray et al., 2018; Hagstrom and Denham, 2018; Hecksteden et al., 2018; Horak et al., 2018; Brennan et al., 2019; Byrd et al., 2019; Castro et al., 2019; Damas et al., 2019a,b; Delgado-Floody et al., 2019; Denham, 2019; Hammond et al., 2019; Haun et al., 2019; Islam et al., 2019; Del Giudice et al., 2020).

*References I-R*:

(Lortie et al., 1984; Prud’homme et al., 1984; Koufaki et al., 2002; Leon et al., 2002; Park et al., 2007; King et al., 2008; McPhee et al., 2010, 2011; Mulroy et al., 2010; Riesco et al., 2010; Karavirta et al., 2011, 2013; Keller et al., 2011; Kacerovsky-Bielesz et al., 2012; Johannsen et al., 2013; Mueller et al., 2015; Pandey et al., 2015; Prestes et al., 2015, 2018; Ross et al., 2015; Leddy et al., 2016; Leifer et al., 2016; Metcalfe et al., 2016; Ogasawara et al., 2016; Raleigh et al., 2016, 2018; Montero and Lundby, 2017; Radnor et al., 2017; da Cunha Nascimento et al., 2018, 2020; Leoñska-Duniec et al., 2018; Mclaren et al., 2018; Mobley et al., 2018; Morton et al., 2018; Peltonen et al., 2018; Kneppers et al., 2019; Massie et al., 2019; Ramírez-Vélez et al., 2019, 2020; Ross L. M. et al., 2019; Krusnauskas et al., 2020; Marsh et al., 2020; Medrano et al., 2020; Nunes et al., 2020; Orssatto et al., 2020; Patel et al., 2021).

*References S-Z*:

(Simoneau et al., 1986; Wilson et al., 1996; Timmons et al., 2005; Wadell et al., 2005; Sisson et al., 2009; Vollaard et al., 2009; Scharhag-Rosenberger et al., 2012; Schmid et al., 2013; Thomaes et al., 2013; Vincent et al., 2014; Sawyer et al., 2015; Stephens et al., 2015, 2018; Wolpern et al., 2015; Stec et al., 2016, 2017; Yalamanchi et al., 2016; Yoo et al., 2016; Schubert et al., 2017; Steele et al., 2017; Stuart et al., 2017; Williamson et al., 2017, 2018; Yan et al., 2017; Valenzuela et al., 2018, 2020; Weatherwax et al., 2018a,b, 2019; Wormgoor et al., 2018; Sandroff et al., 2019; Saric et al., 2019; Seward et al., 2019; Taaffe et al., 2019; Williams et al., 2019; Witvrouwen et al., 2019; Schulhauser et al., 2020; Thomas et al., 2020; Vellers et al., 2020; Walsh et al., 2020; Wang et al., 2020; Yu et al., 2020).
